# Supplementary material for: COZOID: contact zone identifier for visual analysis of protein-protein interactions
Source: BMC Bioinformatics. 2018 Apr 6;19:125. doi: 10.1186/s12859-018-2113-6 (PMC5889581; doi:10.1186/s12859-018-2113-6)
Supplement: Supplementary file 2 — Software build. Executable binary file of the software tool. (ZIP 118,784 kb) [file 12859_2018_2113_MOESM2_ESM.zip › cozoid/platform/docs/testng-6.5.1-javadoc.zip!/org/testng/annotations/ObjectFactory.html]

ObjectFactory (TestNG)


---


|  |  |  |
| --- | --- | --- |
| **Overview** \| **Package** \| **Class** \| **Tree** \| **Deprecated** \| **Index** \| **Help** | |  |
| **PREV CLASS**   **NEXT CLASS** | **FRAMES**    **NO FRAMES**     **All Classes** |  |
| SUMMARY: REQUIRED \| OPTIONAL | DETAIL: ELEMENT |  |


---


## org.testng.annotations Annotation Type ObjectFactory

---

``` @Retention(value=RUNTIME) @Target(value=METHOD) public @interface ObjectFactory ```

Marks a method as the object factory to use for creating all test instances.
The test classes can only contain one method marked with this annotation,
and the method must return an instance of `ITestObjectFactory`.

**Since:**
:   5.6

**Author:**
:   Hani Suleiman

---


|  |  |  |
| --- | --- | --- |
| **Overview** \| **Package** \| **Class** \| **Tree** \| **Deprecated** \| **Index** \| **Help** | |  |
| **PREV CLASS**   **NEXT CLASS** | **FRAMES**    **NO FRAMES**     **All Classes** |  |
| SUMMARY: REQUIRED \| OPTIONAL | DETAIL: ELEMENT |  |


---
